# Case Report: Cross-overlapping multi-purse-string closure of a large gastric wall defect after endoscopic full-thickness resection

**DOI:** 10.3389/fmed.2026.1936608

**Published:** 2026-09-04

**Authors:** Zhiyu Ma, Xuan Bai, Jialong Qi, Qiang Guo, Fan Zhang, Tian He, Zan Zuo

**Affiliations:** 1 Department of Gastroenterology, The First People's Hospital of Yunnan Province, The Affiliated Hospital of Kunming University of Science and Technology, Kunming, China; 2 Yunnan Provincial Clinical Medical Center for Digestive System Diseases, Kunming, China; 3 Department of Gastroenterology, The Third People's Hospital of Yunnan Province, Kunming, China; 4 Yunnan Provincial Key Laboratory of Clinical Virology, The First People's Hospital of Yunnan Province, The Affiliated Hospital of Kunming University of Science and Technology, Kunming, China

**Keywords:** case report, endoscopic full-thickness resection, gastric wall defect, gastrointestinal stromal tumor, nylon loop, purse-string closure

## Abstract

**Background:**

Secure endoscopic closure of large gastric full-thickness defects after endoscopic full-thickness resection (EFTR) remains challenging, particularly when the defect is irregular and dedicated closure devices are unavailable or unsuitable.

**Case presentation:**

A 63-year-old man underwent EFTR for a 4.5-cm gastric body lesion originating from the muscularis propria. Resection created an irregular full-thickness defect of approximately 4 cm. Three nylon loops were independently anchored around the defect margin with metal clips using deliberately staggered, partially overlapping fixation paths. Sequential tightening created a cross-overlapping mesh-like configuration and achieved complete endoscopic closure. Air insufflation showed no leakage. Postoperative imaging demonstrated no significant free air or fluid accumulation, and histopathology confirmed a low-risk gastrointestinal stromal tumor. The patient was discharged on postoperative day 7. Endoscopic follow-up at 3, 6, and 12 months demonstrated progressive mucosal healing without reperforation or stenosis.

**Conclusion:**

Cross-overlapping multi-purse-string closure using conventional nylon loops and metal clips was feasible in this selected large and irregular gastric defect. The technique may offer a device-accessible option when standard single-loop closure is considered insufficient, although its reproducibility and comparative effectiveness require evaluation in larger studies.

## Introduction

Endoscopic full-thickness resection (EFTR) permits *en bloc* removal of selected gastric lesions arising from the muscularis propria while preserving gastric anatomy. However, the intentionally created full-thickness defect must be closed securely to prevent persistent leakage, peritonitis, and intra-abdominal infection (1–3). Conventional through-the-scope clips are useful for small defects but may provide insufficient span or grasping force in large, irregular, or high-tension defects. Purse-string closure using a nylon loop and clips expands the closure range, yet a single loop may concentrate tension along one closure plane (3–5). Over-the-scope clips and dedicated endoscopic suturing systems can provide robust closure but may be limited by defect geometry, access, equipment availability, or cost (1, 6).

We report a cross-overlapping multi-purse-string technique in which three independently anchored nylon loops follow staggered, partially overlapping paths around a large gastric defect. The case is presented because it demonstrates a practical modification of conventional accessories for an irregular approximately 4-cm full-thickness defect, together with 12-month endoscopic follow-up and a detailed procedural video.

## Case description

### Patient information and clinical findings

**Figure 1:**
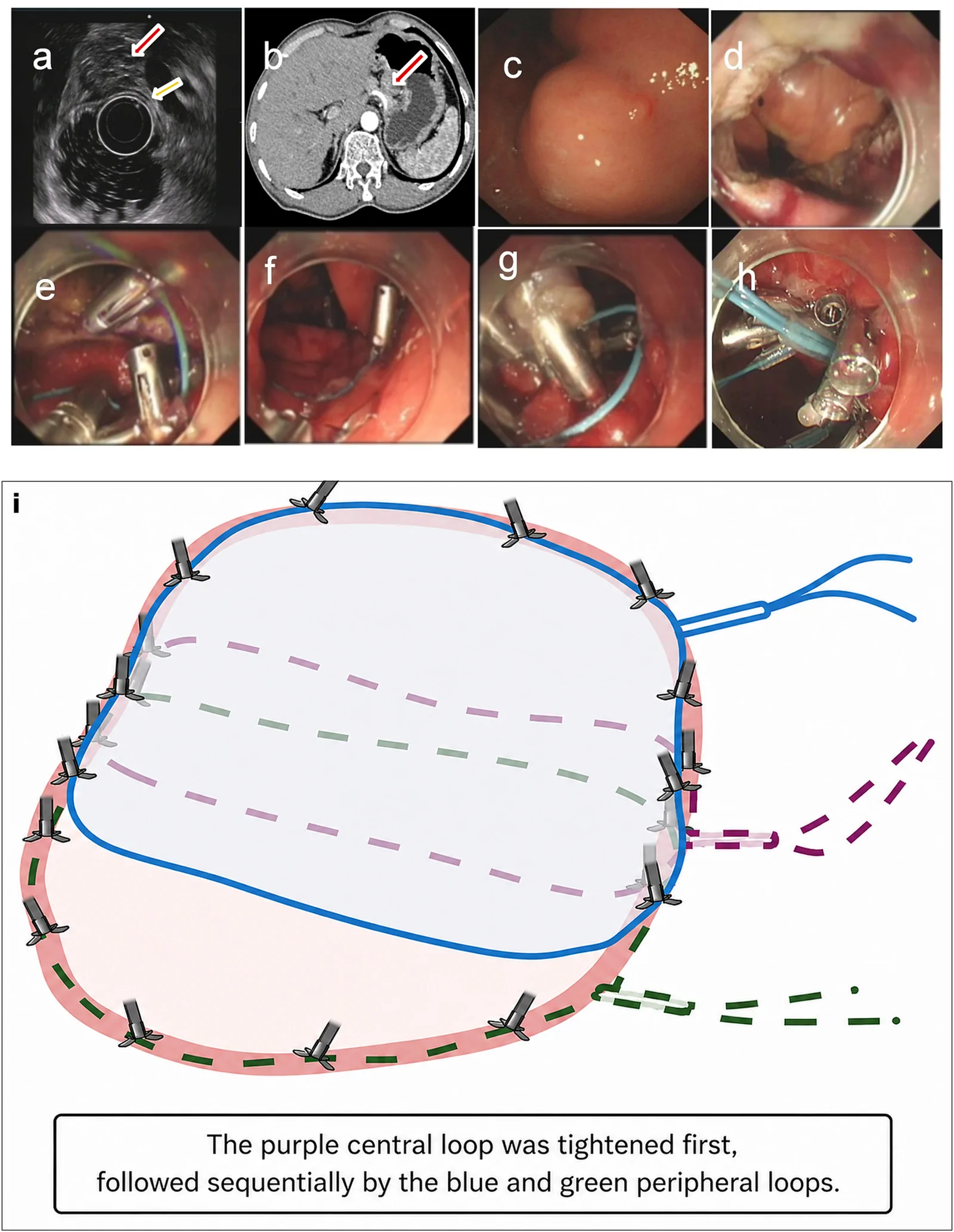
Preoperative assessment and procedural sequence. **(A)** Endoscopic ultrasonography showing a hypoechoic lesion arising from the muscularis propria (red arrow, lesion; yellow arrow, muscularis propria). **(B)** Contrast-enhanced computed tomography showing a gastric body mass without metastasis (red arrow). **(C)** Preoperative endoscopic appearance. **(D)** Irregular full-thickness defect after EFTR. **(E–G)** Sequential anchoring of the first, second, and third staggered nylon loops with metal clips. **(H)** Complete endoscopic closure after sequential tightening. **(I)** Simplified two-dimensional schematic of the three staggered and partially overlapping loop paths before tightening. The purple central loop was tightened first, followed sequentially by the blue and green peripheral loops. Rotatable metal clips anchor the loops. Not to scale.

A 63-year-old man was referred after gastroscopy detected a submucosal lesion in the gastric body. Endoscopic ultrasonography demonstrated a hypoechoic lesion arising from the muscularis propria and measuring approximately 4.5 cm. The identification of muscularis propria origin was relevant to treatment planning and supported the selection of EFTR. Contrast-enhanced abdominal computed tomography showed a gastric soft-tissue mass without evidence of local invasion or metastatic disease (Figures 1A,B). After preoperative multidisciplinary discussion, EFTR with planned endoscopic closure was selected. Written informed consent was obtained for the procedure and for publication of the clinical images and video.

### Diagnostic assessment

**Figure 2:**
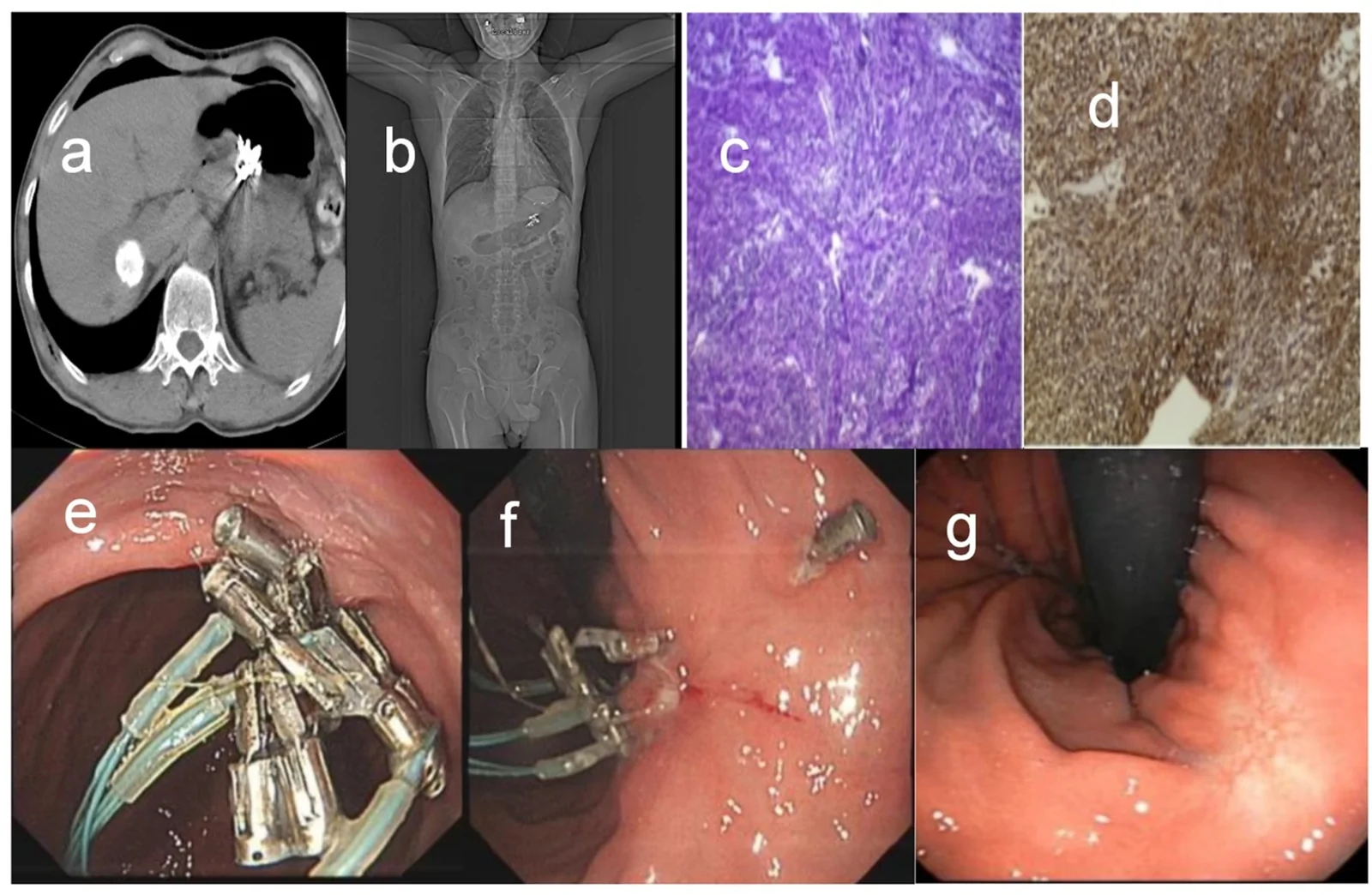
Postoperative assessment, pathology, and follow-up. **(A)** Postoperative abdominal computed tomography showing no significant intra-abdominal free air or fluid accumulation. **(B)** Upright abdominal radiography showing no subdiaphragmatic free air. **(C)** Histopathology showing a spindle-cell tumor. **(D)** Immunohistochemical staining consistent with gastrointestinal stromal tumor. **(E–G)** Follow-up endoscopy at 3, 6, and 12 months, respectively, showing progressive mucosal healing and mature scar-like change.

The lesion was considered a gastric subepithelial tumor originating from the muscularis propria. The absence of metastatic disease on computed tomography supported local endoscopic treatment. The final diagnosis was established after resection: histopathology showed a 4.5-cm spindle-cell tumor with a mitotic count of ≤5 per 50 high-power fields. Immunohistochemical staining was positive for vimentin, CD34, CD117, and DOG-1 and negative for pancytokeratin, desmin, and S-100, consistent with a low-risk gastrointestinal stromal tumor (Figures 2C,D). No tumor rupture was observed. The lesion was removed *en bloc* and was macroscopically completely resected. Imaging showed no regional lymph-node or distant metastasis. According to the eighth edition of the American Joint Committee on Cancer TNM staging system, the tumor was classified as pT2 cN0 cM0, stage IA (7). Following histopathological confirmation, a routine postoperative specialist consultation was obtained. Considering the low-risk pathological features, adjuvant imatinib was not administered, and clinical, radiological, and endoscopic surveillance was selected.

### Therapeutic intervention

EFTR was performed under general anesthesia with endotracheal intubation. Following *en bloc* and macroscopically complete resection, an irregular full-thickness gastric wall defect measuring approximately 4 cm remained (Figures 1C,D). Three nylon loops were delivered sequentially through a single-channel endoscope. Each loop was independently fixed around the defect margin using rotatable metal clips. Rather than placing the loops coaxially, the anchoring points were staggered so that the paths partially overlapped across the defect. This preconfigured a cross-overlapping network before tightening (Figures 1E–G). A total of three nylon loops and 27 rotatable metal clips were used.

After all three loops had been independently anchored, the centrally positioned loop was tightened first, followed by the two peripheral loops. Sequential tightening progressively approximated the defect margins and produced a multilayer, mesh-like apposition rather than relying on a single circumferential loop. Complete closure was achieved (Figure 1H; Supplementary Video 1). Intragastric air insufflation confirmed maintenance of gastric distension without visible gas leakage. The endoscopic treatment time from the initiation of tumor resection to completion of defect closure was approximately 107 min, including approximately 44 min for EFTR and 63 min for defect closure.

### Timeline

**Figure 3:**
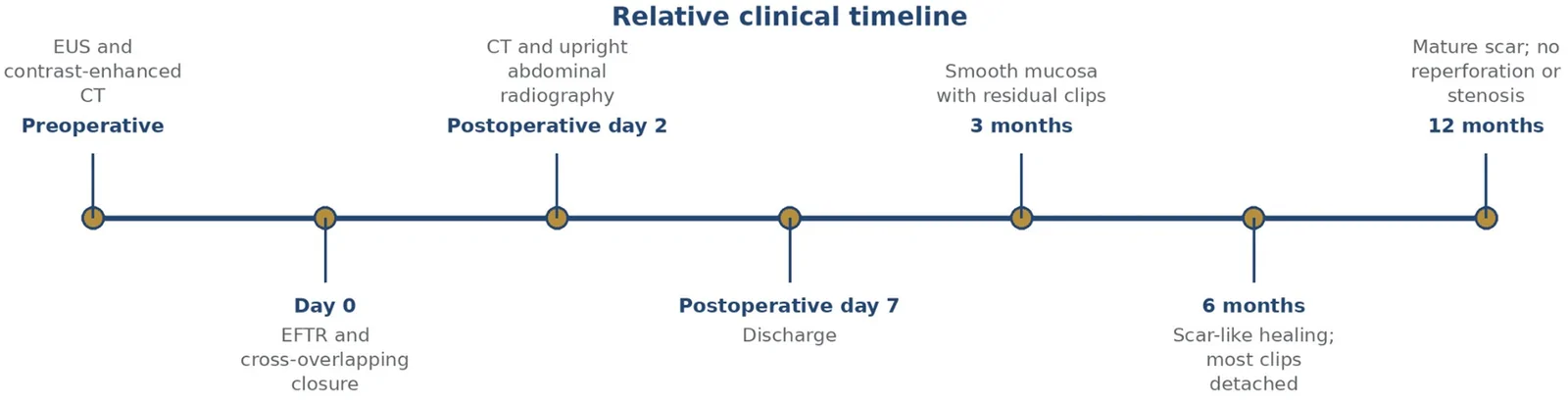
Relative clinical timeline from preoperative assessment through 12-month follow-up.

The relative clinical timeline from preoperative assessment through 12-month follow-up is summarized in Figure 3.

## Follow-up and outcomes

The postoperative course was uneventful. Given the large intentional full-thickness defect, a conservative postoperative management pathway was adopted. The patient remained fasting for four days and received acid suppression, prophylactic broad-spectrum antibiotics, nutritional support, and serial abdominal examinations for signs of leakage, peritonitis, or intra-abdominal infection. Abdominal computed tomography on postoperative day 2 showed no significant intra-abdominal free air or fluid accumulation, while upright abdominal radiography showed no subdiaphragmatic free air (Figures 2A,B). Water and a small amount of liquid diet were initiated on postoperative day 5 and were gradually advanced. The patient did not require repeat endoscopic treatment or surgical intervention. The seven-day hospitalization reflected the institution's conservative post-EFTR observation pathway rather than a complication or other unplanned adverse event. The patient was discharged on postoperative day 7 after remaining asymptomatic and tolerating oral intake.

At 3 months, follow-up gastroscopy showed smooth mucosa at the closure site with residual clips. At 6 months, the site demonstrated scar-like healing and most clips had detached. At 12 months, the defect remained completely healed with mature scar-like change and no evidence of reperforation, ulceration, or stenosis (Figures 2E–G).

Given the low-risk pathological classification, follow-up was planned using a risk-adapted strategy. The 3-, 6-, and 12-month gastroscopies were performed primarily to document local healing at the closure site. Thereafter, oncologic surveillance will include annual abdominal computed tomography for 10 years, consistent with the 2023 GEIS recommendations for low-risk GIST (8), with clinical review at scheduled visits. Additional endoscopy will be performed if clinically indicated or if local reassessment of the resection site is required.

## Discussion

The principal technical feature of this case was the use of several independently anchored loops with staggered and partially overlapping fixation paths. Previous purse-string techniques have demonstrated the usefulness of nylon loops and clips for gastrointestinal wall defects, including large gastric wounds (4, 5, 9). The present configuration differs from a coaxial or repeated single-plane purse-string arrangement: the loops were positioned to overlap spatially before tightening, allowing progressive reduction of an irregular defect from more than one direction. This may reduce dependence on any single loop and may help avoid excessive local tension at one portion of the defect margin. These mechanical explanations remain conceptual because no *ex vivo* tension testing or direct comparison was performed.

Several endoscopic strategies are available for closure of large defects after ESD or EFTR. These include conventional through-the-scope clips, clip-line techniques, clip-loop/purse-string techniques, over-the-scope clips, endoscopic suturing systems such as OverStitch, and helix-tacking systems such as X-Tack. The choice of technique depends on defect size and geometry, tissue quality, anatomic location, device availability, and operator experience (3, 10).

The technique uses accessories commonly available in endoscopy units and does not require a dedicated suturing platform. X-Tack and OverStitch are reasonable alternatives for selected irregular gastrointestinal defects; however, neither system was available at our institution at the time of the procedure. The present approach was selected on the basis of immediate accessory availability, defect geometry, and operator experience, rather than evidence of superiority over X-Tack, OverStitch, over-the-scope clips, or other established closure systems. In this case, the direct accessory cost for defect closure was approximately RMB 2,350, including approximately RMB 1,000 for three nylon loops and RMB 1,350 for 27 rotatable metal clips. At our institution, one over-the-scope clip system costs more than RMB 6,800. These institution-specific costs exclude procedural, personnel, anesthesia, hospitalization, and additional accessory expenses and therefore do not constitute a formal cost-effectiveness analysis. Nevertheless, the procedure required multiple clips and careful planning of loop position. The operator must ensure secure tissue capture, avoid excessive tightening that could tear the defect margin, and confirm closure integrity after sequential loop contraction. The technique should therefore be considered a selected-case option rather than a replacement for established closure systems.

This report has several strengths. It documents the complete procedural sequence, provides a supplementary video, and includes objective postoperative imaging and serial endoscopic follow-up to 12 months. The main limitation is that it describes a single patient treated by an experienced endoscopist. The absence of a control procedure, biomechanical testing, and independent outcome assessment prevents conclusions regarding superiority over double-loop purse-string closure, over-the-scope clips, or endoscopic suturing. In addition, the applicability of the technique to other locations, tissue qualities, and chronic defects is unknown. Prospective multicenter studies and comparative or *ex vivo* evaluations are needed to clarify technical reproducibility, learning requirements, closure strength, adverse events, and cost.

The take-away lesson is that careful spatial planning of conventional loops may expand their utility for selected large and irregular gastric full-thickness defects. The claimed innovation lies in the staggered overlapping anchoring geometry and sequential contraction, not simply in increasing the number of loops.

## Patient Perspective

The patient reported a smooth postoperative recovery and expressed satisfaction that additional surgery had been avoided. He was also satisfied with the sustained healing documented during long-term follow-up.

## Conclusion

In this case, cross-overlapping multi-purse-string closure with three nylon loops and metal clips achieved complete closure of an approximately 4-cm irregular gastric full-thickness defect after EFTR, followed by uneventful recovery and durable healing at 12 months. The method appears feasible for carefully selected defects, but larger comparative studies are required before broader adoption.

## Data Availability

The original contributions presented in the study are included in the article and Supplementary Material. Further inquiries can be directed to the corresponding authors.

## Supplementary material

Supplementary Video 1 Endoscopic full-thickness resection and cross-overlapping multi-purse-string closure of a large gastric wall defect, showing the gastric lesion, EFTR, creation of the full-thickness defect, sequential anchoring of three staggered nylon loops with 27 rotatable metal clips, and tightening of the centrally positioned loop followed by the two peripheral loops to achieve complete endoscopic closure.
